# TGFβ2 mediates oxidative stress–induced epithelial-to-mesenchymal transition of bladder smooth muscle

**DOI:** 10.1007/s11626-024-00864-9

**Published:** 2024-02-26

**Authors:** Jingwen Geng, Xiaofan Zhang, Yansong Zhang, Xiaojia Meng, Jinqi Sun, Bo Zhou, Jun Ma

**Affiliations:** 1 Medical Research Center, The Second Affiliated Hospital of Zhengzhou University, Zhengzhou, 450014 Henan China; 2 Clinical Laboratory, The Second Affiliated Hospital of Zhengzhou University, Zhengzhou, 450014 Henan China

**Keywords:** Benign prostatic hyperplasia, Bladder outlet obstruction, Oxidative stress, Transforming growth factor β2, Epithelial-to-mesenchymal transition

## Abstract

Bladder outlet obstruction (BOO) is the primary clinical manifestation of benign prostatic hyperplasia, the most common urinary system disease in elderly men, and leads to associated lower urinary tract symptoms. Although BOO is reportedly associated with increased systemic oxidative stress (OS), the underlying mechanism remains unclear. The elucidation of this mechanism is the primary aim of this study. A Sprague–Dawley rat model of BOO was constructed and used for urodynamic monitoring. The bladder tissue of rats was collected and subjected to real-time reverse transcription-quantitative polymerase chain reaction (RT-qPCR), histological examination, and immunohistochemical staining. Through bioinformatics prediction, we found that transforming growth factor β2 (TGFβ2) expression was upregulated in rats with BOO compared with normal bladder tissue. In vitro analyses using primary bladder smooth muscle cells (BSMCs) revealed that hydrogen peroxide (H_2_O_2_) induced TGFβ2 expression. Moreover, H_2_O_2_ induced epithelial-to-mesenchymal transition (EMT) by reducing E-cadherin, an endothelial marker and CK-18, a cytokeratin maker, and increasing mesenchymal markers, including N-cadherin, vimentin, and α-smooth muscle actin (α-SMA) levels. The downregulation of TGFβ2 expression in BSMCs using siRNA technology alleviated H_2_O_2_-induced changes in EMT marker expression. The findings of the study indicate that TGFβ2 plays a crucial role in BOO by participating in OS-induced EMT in BSMCs.

**Supplementary Information:**

The online version contains supplementary material available at 10.1007/s11626-024-00864-9.

## Introduction

Benign prostatic hyperplasia (BPH) is the most common urinary system disease in older men. It affects approximately 30–40% of men aged ≥ 40 yr and 70–80% of men aged ≥ 80 yr (Madersbacher *et al.* 2019). The disease primarily manifests as lower urinary tract symptoms, including frequent urination, urinary urgency, and urinary hesitancy. Currently, oral medications are the gold standard for the clinical treatment of BPH. However, combination therapy using receptor blockers and 5α-reductase inhibitors has shown superior effects to monotherapy or placebo and can considerably delay the clinical progression of BPH. Nevertheless, approximately 12.6% of male patients receiving combination therapy continue to experience varying degrees of progression up to 4 yr after treatment (Roehrborn 2011). The lack of a therapeutic response or persistent symptoms has prompted an in-depth investigation of the pathophysiological mechanisms underlying BPH (Thomas-White *et al*. 2016).

BPH causes nodular hyperplasia in prostate tissue and can induce various symptoms, including bladder outlet obstruction (BOO) (Lloyd *et al*. 2019), the primary clinical manifestation of BPH. The ever-expanding aging global population has led to an increase in the incidence of BOO (He *et al*. 2022). Furthermore, long-term BOO can cause changes in bladder function and structure, involving inflammation and hypertrophy, hyperplasia, and decompensated fibrosis of bladder smooth muscle (Wang *et al*. 2021). Currently, BOO-mediated bladder smooth muscle proliferation mechanisms remain unclear, and there are no effective treatment methods to prevent or reverse bladder remodelling (Chen *et al*. 2020).

Cyclic ischaemia/reperfusion is the primary aetiology of bladder dysfunction caused by BOO (Greenland and Brading 2001). Bladder ischaemia/reperfusion injury generates reactive oxygen species (ROS), such as superoxide anions and hydroxyl radicals, leading to the peroxidation of cellular and subcellular membranes (Matsumoto and Kakizaki 2012). Oxidative stress (OS) refers to the pro-oxidant state arising from an imbalance between oxidant and antioxidant effects in the body. An increase in OS can cause cell damage and transformation. The induced cellular effects primarily arise through protein and lipid modifications, which alter original functions and accelerate abnormal cellular processes (Montorfano *et al*. 2014). Moreover, OS results in neutrophilic inflammatory infiltration, increased protease secretion, and the formation of numerous oxidation intermediates. OS is associated with an increase in the level of intracellular ROS, which is typically generated at low levels during the redox reaction of water and has crucial roles in myriad biological processes, including apoptosis, immunity, cellular defence against microorganisms, and bladder dysfunction (Kurutas *et al*. 2005; Miyata *et al*. 2019). Additionally, ROS may cause damage to mitochondria in the detrusor muscle, thereby inhibiting energy production and impairing detrusor contractility (Lin *et al*. 2005). Adequate blood supply is necessary for acquiring oxygen and nutrients required for normal organ operation, whereas ischaemia impairs organ function. Reduced blood flow and chronic bladder ischaemia exist in many animal models of BOO. Therefore, a comprehensive understanding of the pathological significance of OS in bladder dysfunction and BOO is needed to understand the development of effective treatment strategies.

Oxidative stress can induce epithelial-to-mesenchymal transition (EMT), a common cellular transformation mechanism through which epithelial cells alter their phenotype and functional characteristics. This leads to the loss of epithelial features and simultaneous gain of mesenchymal characteristics, causing a transformation into myofibroblasts and promoting the occurrence of tissue fibrosis (Montorfano *et al*. 2014; Wang *et al*. 2017). The occurrence of EMT in epithelial cells is followed by changes in protein expression profiles. Specifically, the expression of the epithelial marker E-cadherin is downregulated, whereas that of fibroblast-specific genes, such as N-cadherin and α-smooth muscle actin (α-SMA), is upregulated (Ishisaki *et al*. 2003; Krenning *et al*. 2008; Medici *et al*. 2011). The downregulation of E-cadherin is an initial and key step in EMT. However, this process is inhibited by various transcription factors, including Snail, Slug, Twist, and ZEB1 (Polyak and Weinberg 2009). Research has shown that epithelial cells not only undergo EMT to transform into myofibroblasts but are also capable of synthesising extracellular matrix (ECM) components, such as collagen I and fibronectin (Li *et al*. 2016). Moreover, human epidermal keratinocytes stimulated by H_2_O_2_ treatment exhibit protein expression changes identical to those of EMT. Similar results have been observed in renal tubular epithelial cells ( Rhyu *et al*. 2005; Fukawa *et al*. 2012).

OS is closely associated with various complex physiological and pathological mechanisms (Pizzino *et al*. 2017; Andrisic *et al*. 2018). An increase in ROS promotes cell proliferation, apoptosis, angiogenesis, and migration under physiological conditions (Brown and Griendling 2015). More importantly, excessive ROS production causes OS, which can damage cellular lipids, proteins, and DNA (Trachootham *et al*. 2009; Schieber and Chandel 2014). An increasing number of studies have also demonstrated the importance of OS in carcinogenesis, malignant behaviors, and the prognosis of various types of cancers (Carini *et al*. 2017).

Low levels of OS may promote TGFβ2-induced EMT of human retinal pigment epithelial cells (Yang *et al*. 2020). However, little is known about the molecular mechanism of TGFβ2 in the development of bladder fibrosis following BOO. The purpose of this study is to determine whether TGFβ2 serves a crucial role in BOO by participating in OS-induced EMT in BSMCs. Understanding the root causes of bladder dysfunction is essential for developing potential treatment strategies.

## Materials and methods

### Analysis of Gene Expression Omnibus data

Transcriptome sequencing data or microarray chip data associated with rat BOO were screened from the National Center for Biotechnology Information’s Gene Expression Omnibus (GEO) database. Raw data corresponding to obstruction for 10 d, 6 wk, and 4 wk were separately analysed utilising the GSE47080 and GSE167430 datasets. Differentially expressed genes (DEGs) were screened based on fold change ≥ 2 or ≤ 0.5 and *P* < 0.05 criteria.

### Establishment of a BOO rat model

For obstruction studies, 6-wk-old female Sprague–Dawley rats were purchased from Henan Huaxing Laboratory Animal Center (Zhengzhou, China) and housed under pathogen-free conditions. The rats were randomly divided into two groups and reared in a controlled environment (temperature, 21–25°C; humidity, 45–55%). Each rat was anaesthetised via intraperitoneal injection and secured onto the surgical table in a supine position. The surgical site was disinfected, and an incision approximately 1 cm long was made along the midline of the lower abdomen. Subsequently, a blunt dissection was performed, and the bladder was exposed. The experimental group underwent ligation of the bladder neck using a 3–0 surgical suture, whereas the sham surgery group underwent suturing after bladder exposure. Urodynamic measurements were taken for the BOO and control rats 6 wk after surgery, and the rats were subsequently sacrificed. All animal studies were reviewed and approved by the Institutional Ethics Committee of Zhengzhou University (2022070).

### Urodynamic experiment

Each rat received 10% urethane (1 g/kg) via intraperitoneal injection and was secured onto the surgical table in a supine position upon reaching an appropriate level of anaesthesia. An epidural catheter for anaesthesia (1 mm in diameter) was coated with paraffin oil and slowly inserted into the bladder to a depth of 3–4 cm. Urine was aspirated from the bladder using a 1-mL syringe. A three-way stopcock was connected to a bladder pressure measurement tube, bladder pressure sensor, and microinfusion pump. After the connections were made, the air in the various tubing was removed. Monitoring was performed using a urodynamic analyser system (Laborie urodynamic system, Montreal, Canada). After the pressure measurement software had been zeroed, room temperature saline was injected into the rat bladder at a rate of 0.2 mL/min using the microinfusion pump. During the injection process, the urethral orifice and pressure measurements made by the instrument were closely monitored. The recorded parameters included intravesical pressure.

### Immunohistochemistry

The bladder was removed from each rat, rinsed with saline, and divided into three equal sections. Each section was placed in a 4% paraformaldehyde solution for fixation and preservation. After 48 h, the occurrence of protein denaturation and coagulation was observed. The bladder tissue was embedded in paraffin and sectioned into 4-μm-thick slices for staining. Haematoxylin and eosin staining (Beyotime, Shanghai, China) was performed for the observation of gross morphology. Masson’s trichrome and Sirius Red staining were performed to evaluate the degree of fibrosis. A PANNORAMIC MIDI scanner (3DHISTECH, Budapest, Hungary) was used to obtain the images. The collagen fibres of Masson’s trichrome–stained tissue appeared blue, whereas muscle fibres, fibrous proteins, and red blood cells appeared red. In Sirius Red–stained tissue, collagen fibres appeared red on a yellow background.

### Isolation, culture, and identification of primary BSMCs

BSMCs were obtained using the collagenase digestion method. In brief, rats were anaesthetised using the method described earlier, and the bladder was excised after the removal of surrounding adipose and connective tissue. Mucosal and muscular layers were dissected using dissecting scissors under a dissecting microscope. The rat bladder was placed in 0.25% trypsin and incubated at 37°C for 30 min. Subsequently, the bladder tissue was cut into small fragments and incubated in 5 mg/mL type IV collagenase at 37°C for 60 min. The resultant cell suspension was centrifuged at 1000 rpm for 5 min, and the cells were collected. BSMCs were cultured in DMEM/F12 medium (Biological Industries, Beit HaEmek, Israel) supplemented with 10% foetal bovine serum (Gibco, Grand Island, NY), 100 U/mL penicillin, 100 mg/mL streptomycin, and growth additives at 37°C in a 5% CO_2_ environment. Second- and third-passaged cells were used for the experiments.

Cells were identified by immunofluorescence staining of α-SMA, and approximately 95% of the cells were BSMCs. Immunofluorescence testing was performed when the cells reached approximately 90% confluence. Cells were incubated with an anti-α-SMA antibody (14,395–1-AP, Proteintech, Chicago, IL) in a wet box overnight at 4°C. The following day, the primary antibody was removed, and a species-specific secondary antibody, FITC Donkey anti-rabbit IgG (BioLegend, San Diego, CA), was added. After rinsing and DAPI staining, the cells were mounted with an anti-fade mounting medium. Microphotographs were taken using a ZEISS Axiocam 305 colour camera and ZEISS Axio Observer 3 microscope (ZEISS ZEN blue edition Software, version 3.3, Jena, Germany).

To mimic the oxidative stress condition, we conducted pre-experiments to determine the concentration gradient. Cells were treated with 0, 10, 20, 50, 100, 200, 300, and 500 μM of H_2_O_2_ (Sigma, St. Louis, MO), and 100 μM was identified as the optimal concentration.

### Small interfering RNAs and transfection experiments

Small interfering RNAs (siRNAs) were synthesised by GenePharma (Shanghai, China) and transfected using Lipofectamine 2000 (Life Technologies, Carlsbad, CA), to obtain a final concentration of 50 nM according to the manufacturer’s instructions. At 48 h after transfection, the cells were collected for subsequent analysis. Supplementary Table S1 shows the siRNA sequences used in this study.

### Reverse transcription-quantitative polymerase chain reaction

Total RNA was extracted with TRIzol reagent (Beyotime) and reverse transcribed to cDNA using the HiScript II Reverse Transcriptase Kit (Vazyme Biotech, Nanjing, China) according to the manufacturer’s instructions. Quantitative polymerase chain reaction (qPCR) was performed on a 7500 real-time PCR system (Applied Biosystems, Foster City, CA) using the Taq Pro Universal SYBR qPCR Master Mix (Vazyme Biotech) according to the manufacturer’s instructions. The mRNA level of glyceraldehyde-3-phosphate dehydrogenase (*GAPDH*) was used as a reference. Supplementary Table S2 shows the primer sequences used for qPCR.

### Western blotting

Cells were lysed in RIPA buffer, and the total protein content was quantified using the BCA Protein Assay Kit (Beyotime, Jiangou, China). Total denatured protein (30 μg) was subjected to sodium dodecyl sulphate–polyacrylamide gel electrophoresis (SDS-PAGE) and transferred onto a polyvinylidene difluoride (PVDF) membrane (Millipore, Billerica, MA). The protein bands were incubated with the primary antibody overnight at 4°C and subsequently with horseradish peroxidase (HRP)-conjugated secondary antibody for 1 h at room temperature. Immunocomplexes were detected using the SuperSignal West Pico Chemiluminescent Substrate (Thermo Fisher Scientific, Waltham, MA). The primary antibodies anti-TGFβ2 (19,999–1-AP), anti-N-cadherin (22,018–1-AP), and anti-α-SMA (14,395–1-AP) were obtained from Proteintech. The anti-E-cadherin (ab231227) and anti-vimentin (ab92547) antibodies were obtained from Abcam (Cambridge, UK). The anti-CK-18 antibody (AF0191) was obtained from Affinity (Affinity Biosciences, Cincinnati, OH), and the anti-GAPDH antibody (#2118) was obtained from Cell Signaling Technology (Danvers, MA).

### Statistical analysis

Data were expressed as mean ± standard deviation (SD) and analysed with GraphPad Prism 9.0 (GraphPad Software, La Jolla, CA). Each experiment was performed at least thrice unless otherwise stated. Statistical significance analysis was performed using a two-tailed Student’s *t*-test. Differences were considered statistically significant at *P* < 0.05.

## Results

### TGFβ2 is frequently upregulated in BOO rats

**Figure 1.**
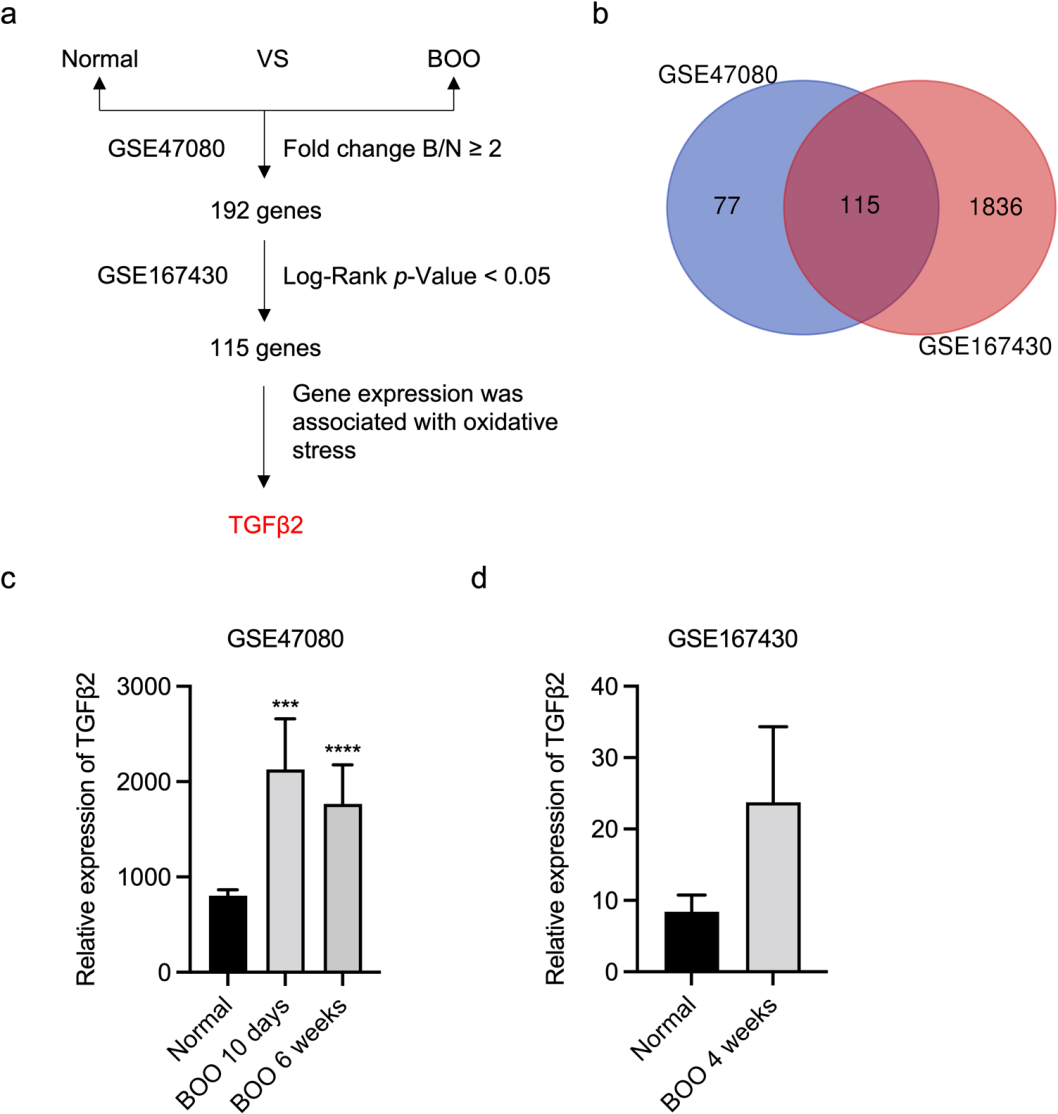
TGFβ2 expression is upregulated in the bladders of bladder outlet obstruction (BOO) rats. (*a*) Schematic representation of upregulated genes associated with oxidative stress (OS) in rats with BOO screened from the GEO database. (*b*) Differentially expressed genes were identified in BOO rats compared with their corresponding controls in GSE47080 and GSE167430 datasets (fold change ≥ 2 or ≤ 0.5, *P* < 0.05). (*c*) Expression of TGFβ2 in bladder tissues of rats obstructed for 10 d and 6 wk compared with control rats in the GSE47080 dataset. (*d*) Expression of TGFβ2 in bladder tissues of rats obstructed for 4 wk compared with control rats in the GSE167430 dataset. The data are presented as the mean ± SD; ***P < 0.001, ****P < 0.0001 using the Student’s *t*-test.

Gene expression data of BOO rats was downloaded from the GEO database to identify new genes involved in BOO development. A total of 115 DEGs (fold change ≥ 2 or ≤ 0.5, *P* < 0.05) were screened via combined analysis (Fig. 1*a*, *b*). GREM1, SGCG, CILP, ACTA1, ADPRHL1, GLRA1, PRC1, PRSS35, FXYD6, and ANO4 were the Top 10 upregulated genes based on GEO analysis. Additionally, downregulated genes, such as SULT1A1, IGFBP3, CCL11, NMBR, NEFL, MAMDC2, IRF7, OAS1B, SLITRK6, and OAS1A, were also identified. Supplementary Table S3 presents the fold change values for each gene. In the BOO group, rats exhibited BSMC hypertrophy and interstitial cell proliferation during the first 4 wk following BOO onset. Moreover, TGFβ2 was upregulated in the bladder of rats after 10 d, 6 wk (Fig. 1*c*), and 4 wk (Fig. 1*d*) of BOO. Given its association with the OS pathways, TGFβ2 was selected for further investigation.

### Histological characteristics and urodynamic changes in BOO rats

**Figure 2.**
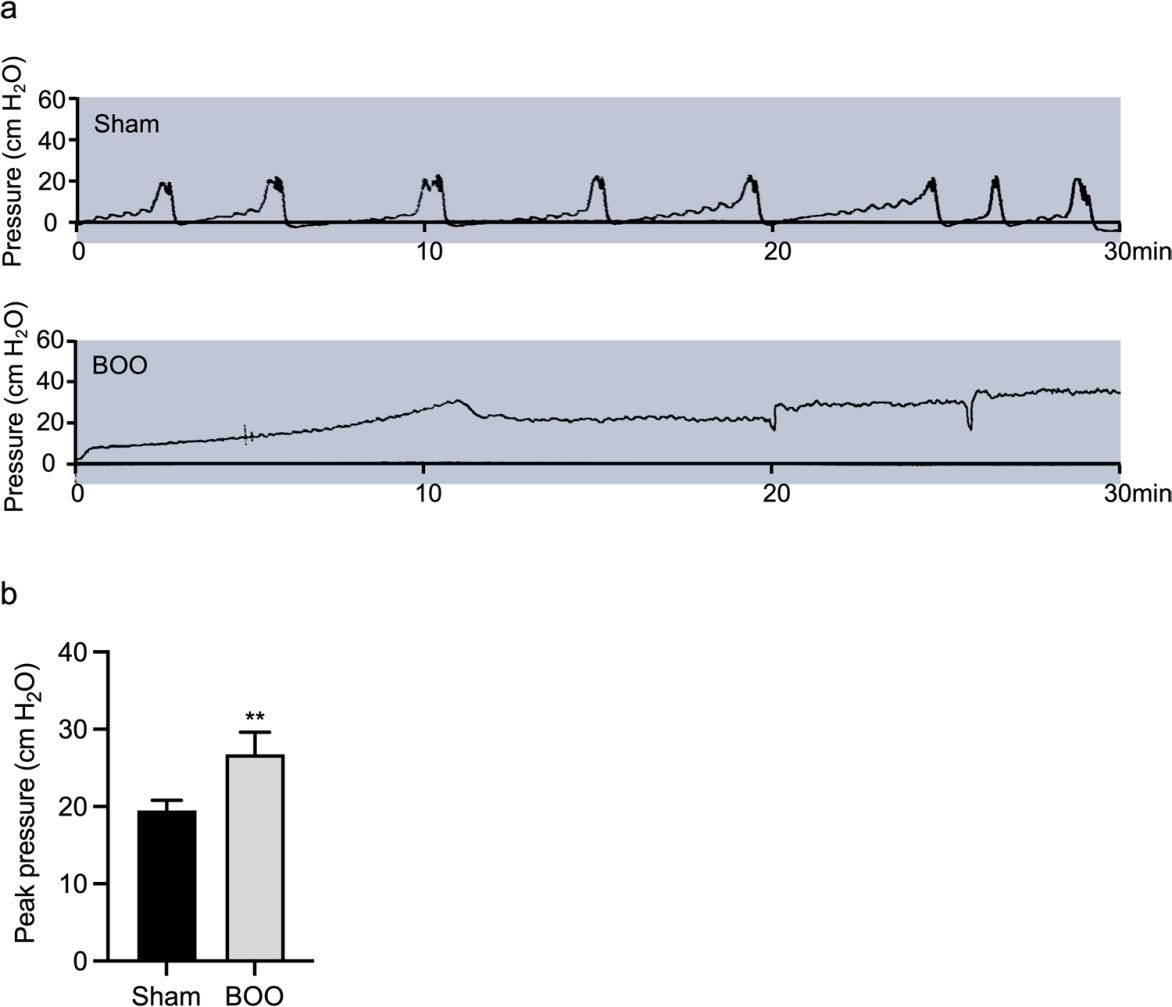
Urodynamic assessment of control and BOO rats. (*a*) Representative continuous cystometry recordings of control and 6-wk-old BOO rats. (*b*) Urodynamic parameters evaluated peak bladder pressure. The data are presented as the mean ± SD; **P < 0.01 using the Student’s *t*-test.

Urodynamic assessment was performed on anaesthetised rats to confirm that BOO caused bladder dysfunction (Fig. 2*a*, *b*). The peak bladder pressure during voiding was 18–20 cm H_2_O in the control group, and 26–31 cm H_2_O in the 6-wk BOO group; i.e., the BOO group exhibited a significant increase in peak voiding pressure compared with the control group.

**Figure 3.**
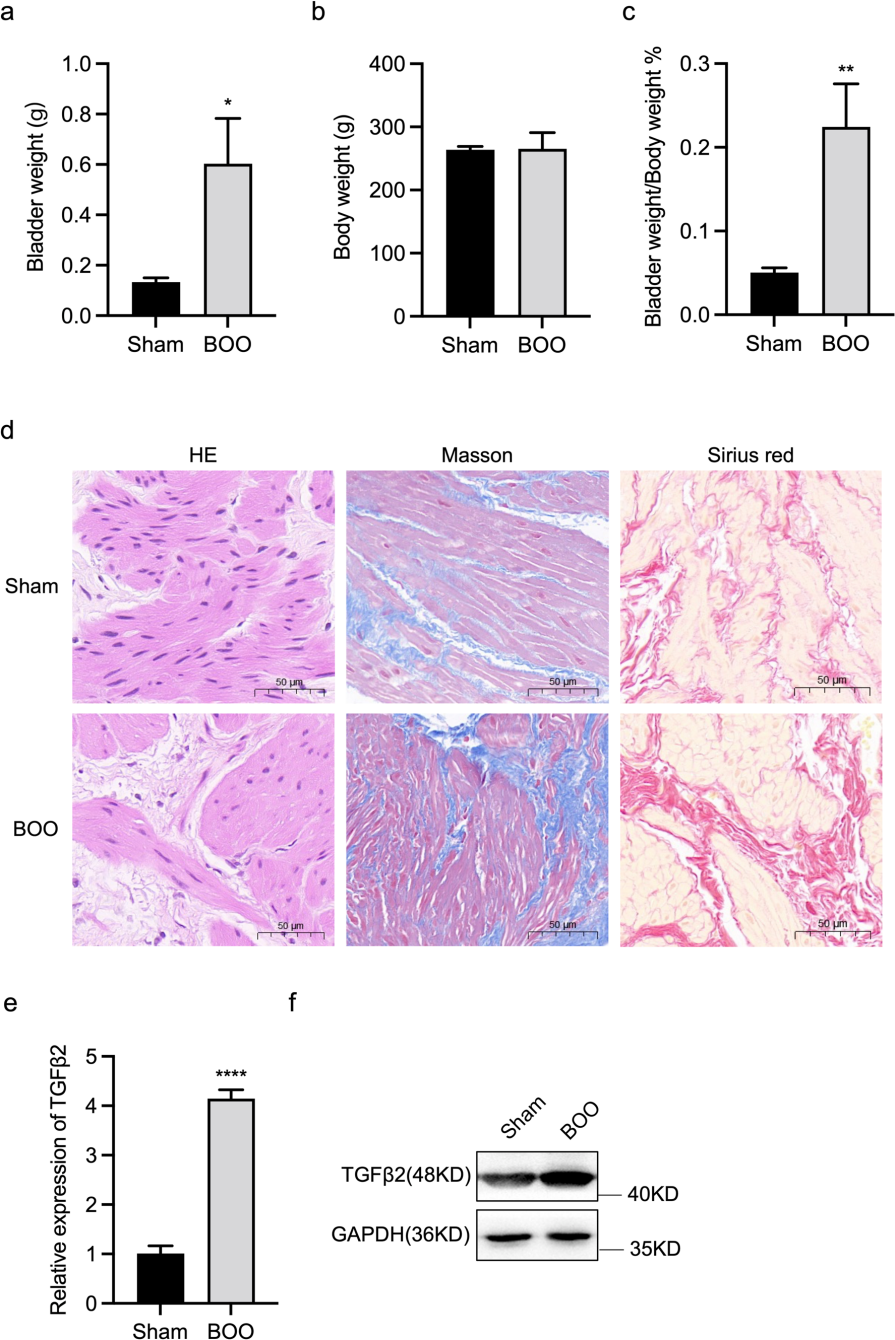
Histological features of control and BOO rats. (*a*) Bladder weight, (*b*) body weight, and (*c*) bladder weight to body weight ratio of control and 6-wk-old BOO rats. (*d*) Left: HE staining. *Middle*: Masson’s trichrome staining. *Magnification* 200 × . *Red* areas indicate smooth muscle, and *blue* areas indicate connective tissue. In the control group, only a small amount of pale blue collagen deposition was observed between muscle bundles, and the 6-wk-old BOO group showed increased collagen deposition and connective tissue. *Right*: Sirius Red staining, swollen smooth muscle (*light yellow*), and increased collagen fibres (*red*) appeared in the 6-wk-old BOO group. The (*e*) mRNA and (*f* ) protein expression levels of TGFβ2 in bladder tissue of rats in the control and 6-wk-old BOO groups. The data are presented as the mean ± SD; *P < 0.05, **P < 0.01, ****P < 0.0001 using the Student’s *t*-test.

No significant differences were observed in preoperative body weight among the various groups. All rats remained healthy and survived until the experimental endpoint. However, rats in the 6-wk BOO group experienced a significant increase in bladder weight compared with the control group (Fig. 3*a*). Moreover, body weight analysis showed no significant differences between the BOO and control groups at 6 wk (Fig. 3*b*). This resulted in a significant increase in the bladder weight/body weight ratio in the BOO group (Fig. 3*c*).

To further determine whether BOO led to bladder wall hypertrophy and fibre formation, we performed HE, Masson’s trichrome, and Sirius Red staining to facilitate histological analysis. The bladder tissue of the control group only exhibited sparse light-blue staining of collagen fibres between the smooth muscle bundles. In the BOO group, the degree of collagen fiber deposition and interstitial tissue fibrosis increased significantly (Fig. 3*d*).

To elucidate the role of TGFβ2 in the development of bladder fibrosis following BOO, its expression was quantified. Reverse transcription-qPCR (RT-qPCR) and western blotting results showed that TGFβ2 mRNA and protein expression in the bladders of 6-wk BOO rats were significantly higher than those of the control group (Fig. 3*e*, *f*). These results demonstrate that TGFβ2 participates in BOO-induced bladder fibrosis.

### BOO promotes the expression of EMT-associated genes in the bladder tissue

**Figure 4.**
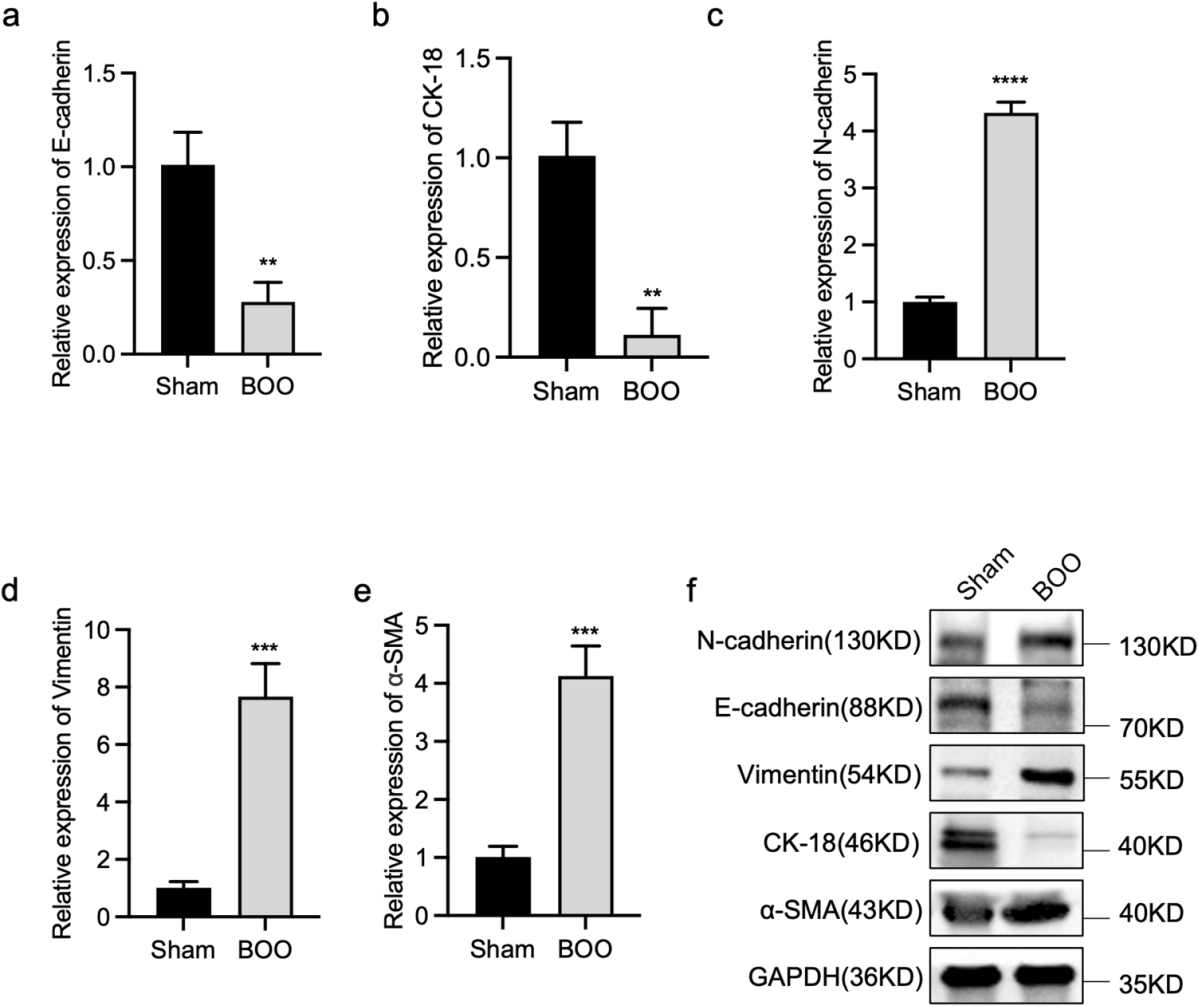
Expression of E-cadherin, CK-18, N-cadherin, vimentin, and α-SMA in bladder tissue of control and BOO rats. The mRNA expression levels of (*a*) E-cadherin, (*b*) CK-18, (*c*) N-cadherin, (*d*) vimentin, and (*e*) α-SMA were detected using RT-qPCR. ( *f* ) The protein expression levels of E-cadherin, N-cadherin, vimentin, CK-18, and α-SMA were detected using western blotting. The data are presented as the mean ± SD; **P < 0.01, ***P < 0.001, ****P < 0.0001 using the Student’s *t*-test.

Considering the BOO-induced thickening of bladder epithelium and the importance of EMT in the epithelial barrier, we detected changes in EMT-associated genes in bladder tissue samples from BOO and control rats using RT-qPCR and western blotting. When compared with the control group, the presence of E-cadherin and CK-18 was significantly decreased in the BOO group (Fig. 4*a*, *b*). The bladder tissue was also tested for mesenchymal markers (N-cadherin, vimentin, and α-SMA). BOO substantially increased the expression of N-cadherin, vimentins, and α-SMA in the bladder epithelium compared with the control group (Fig. 4*c*-*e*). These results were consistent with the RT-qPCR results, i.e., the decrease in E-cadherin and CK-18 protein expression and the increase in N-cadherin, vimentin, and α-SMA protein levels in the BOO group (Fig. 4*f*). These data imply that EMT occurs in the bladder tissue of BOO rats.

### OS-induced EMT is dependent on TGFβ2 expression

First, primary rat BSMCs were isolated and cultured. Immunofluorescence staining of α-SMA confirmed the successful isolation and culture of primary BSMCs, with the proportion of BSMCs in the cell culture exceeding 95% (Supplementary Fig. S1). Next, TGFβ2-targeting siRNA was used to inhibit TGFβ2 expression in primary BSMCs. RT-qPCR and western blotting revealed that the knockdown efficiency of the siRNA was > 90% (Supplementary Fig. S2*a*, *b*).

**Figure 5.**
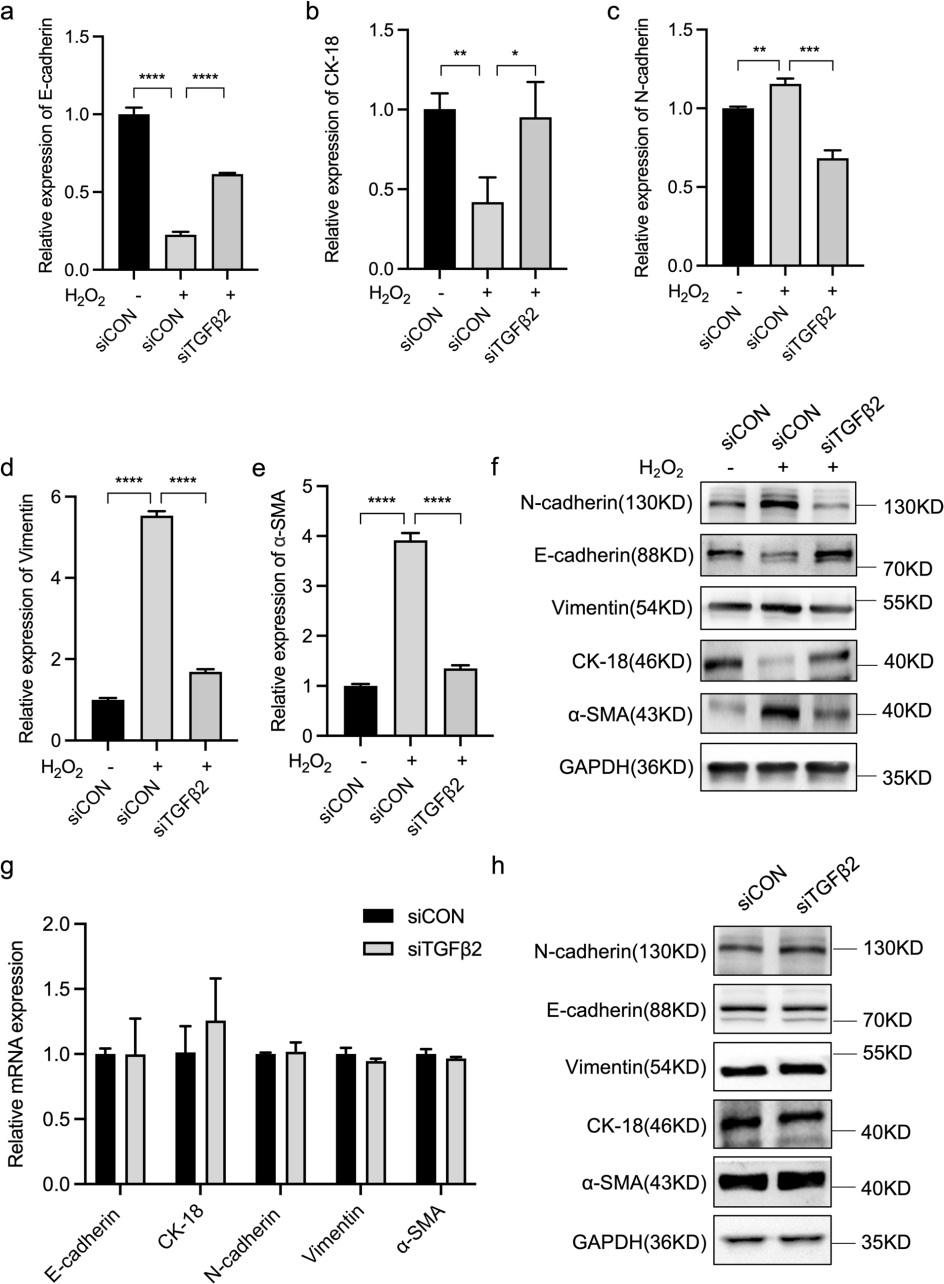
TGFβ2 inhibits OS-induced EMT in BSMCs. BSMCs transfected with siRNA targeting TGFβ2 (siTGFβ2) or non-targeting siRNA (siCON) were incubated for 24 h in the absence (–) or presence ( +) of 100 μM H_2_O_2_. The mRNA expression levels of (*a*) E-cadherin, (*b*) CK-18, (*c*) N-cadherin, (*d*) vimentin, and (*e*) α-SMA were detected using RT-qPCR. (*f* ) The protein expression levels of E-cadherin, N-cadherin, vimentin, CK-18, and α-SMA were detected using western blotting. BSMCs transfected with siTGFβ2 or siCON in the absence of H_2_O_2 _were used to measure the mRNA and protein expression levels of E-cadherin, N-cadherin, CK-18, vimentin, and α-SMA using (*g*) RT-qPCR and (*h*) western blotting. The data are presented as the mean ± SD; *P < 0.05, **P < 0.01, ***P < 0.001, ****P < 0.0001 using the Student’s *t*-test.

We measured the mRNA and protein expression levels of several known epithelial and mesenchymal markers in H_2_O_2_-treated primary BSMCs to investigate the potential correlations among these factors. BSMCs exposed to 100 μM H_2_O_2_ exhibited an increase in TGFβ2 levels (Supplementary Fig. S3*a*, *b*). BSMCs treated with H_2_O_2_ and transfected with non-targeting siRNA were used as the control (siCON). RT-qPCR results showed a significant decrease in E-cadherin and CK-18 mRNA expression in these cells (Fig. 5*a*, *b*), whereas that of N-cadherin, vimentin, and α-SMA was significantly elevated compared with the control group (Fig. 5*c*-*e*). In contrast, cells treated with H_2_O_2_ and transfected with siTGFβ2 exhibited a significant increase in E-cadherin and CK-18 mRNA expression levels (Fig. 5*a*, *b*). Furthermore, transfection with siTGFβ2 prevented the increase in N-cadherin, vimentin, and α-SMA mRNA expression (Fig. 5*c*-*e*). These results indicate that TGFβ2 expression plays a key role in the H_2_O_2_-induced transformation of epithelial cells into myofibroblasts.

To further validate these findings, western blotting was performed to assess the abundance of E-cadherin, N-cadherin, vimentin, CK-18, and α-SMA proteins. The results were consistent with the RT-qPCR results, indicating that TGFβ2 knockdown significantly reversed the decrease in E-cadherin and CK-18 protein expression and the increase in N-cadherin, vimentin, and α-SMA protein levels caused by H_2_O_2_ treatment (Fig. 5*f*). Collectively, these results suggest that OS-induced EMT in BSMCs is dependent on TGFβ2 expression.

### TGFβ2 does not influence EMT in primary BSMCs in the absence of OS

Previous research reported that TGFβ2 is the most effective inducer of EMT and can regulate the expression of EMT-associated genes (Sabbineni *et al*. 2018). Therefore, we investigated whether TGFβ2 downregulation in BSMCs alters the expression levels of EMT-associated genes in the absence of an oxidant. BSMCs transfected with non-targeting siRNA were used as a control (siCON). The mRNA and protein expression levels of E-cadherin, N-cadherin, CK-18, vimentin, and α-SMA were not significantly impacted by TGFβ2 knockdown (Fig. 5*g*, *h*). These findings indicate that epithelial cells and mesenchymal markers did not undergo significant changes when solely subjected to TGFβ2 knockdown. Therefore, the EMT process of primary BSMCs was unaffected. This further confirms that the H_2_O_2_-induced transformation of epithelial cells into myofibroblasts is dependent on TGFβ2 expression.

## Discussion

A de-obstructed rat bladder is not an organ that falls between a normal bladder and an obstructed bladder. On the contrary, the de-obstructed bladder exhibits unique gene expression, morphology, and functional characteristics at the single-cell level, and its tissue structure differs significantly from the control and obstructed bladder groups (Uvelius and Andersson 2022). From a structural perspective, the stretching of BSMCs initiates a growth process that includes protein and DNA synthesis (Saito *et al*. 1994; Zeidan *et al*. 2000). In our bioinformatics analysis of the GEO dataset (Barrett *et al*. 2013), we observed that many mRNAs exhibited increased expression in the obstructed bladder, which subsequently decreased to normal levels following de-obstruction. However, the expression levels of other mRNAs were reduced in the obstructed bladder but increased after de-obstruction. Moreover, certain mRNAs exhibited significant upregulation or downregulation in the de-obstructed bladder (Uvelius and Andersson 2022). Expression of *TGFβ2* mRNA was significantly increased in the obstruction group but reverted to normal in the de-obstructed bladder. This suggests that TGFβ2 plays a certain role in the process of BOO and may serve as a potential therapeutic target for BOO-induced fibrosis. However, the precise mechanisms of action have not yet been thoroughly investigated.

In mammals, TGFβ exists in three isoforms, namely TGFβ1, TGFβ2, and TGFβ3. These isoforms serve crucial roles in cell differentiation, tissue development, wound healing, immune regulation, and tissue fibrosis during immune dysregulation (Sun *et al*. 2021). Previous studies have demonstrated that TGFβ isoforms are expressed during different stages of fibrotic diseases (Querfeld *et al*. 1999; Burke *et al*. 2016; Dropmann *et al*. 2016; Shin *et al*. 2019), with TGFβ1 considered a key mediator of bladder remodelling during BOO progression and a potent regulator of cellular phenotypes in fibrotic diseases (Leask and Abraham 2004; Duan *et al*. 2015). The use of TGFβ1/Smad pathway inhibitors has been reported for the treatment of fibrosis in organs such as the liver, lungs, heart, and kidneys (Jiang *et al*. 2015; Lu *et al*. 2017). However, there is little evidence demonstrating the direct involvement of TGFβ2 in tissue fibrosis. In the present study, combined bioinformatics and experimental analysis revealed that TGFβ2 expression was significantly increased in the BOO model. Following the establishment of an OS environment to stimulate BSMCs, epithelial marker expression was increased, whereas mesenchymal marker expression was decreased, thereby inducing EMT. Moreover, TGFβ2 downregulation eliminated the changes in epithelial and mesenchymal marker expression induced by H_2_O_2_. However, TGFβ2 downregulation alone was not sufficient to affect the expression levels of these markers. Therefore, we deduced that the decrease in TGFβ2 expression level might contribute to the inhibition of OS-induced EMT in BSMCs, which leads to the alleviation of bladder fibrosis.

OS serves a crucial role in the pathological mechanisms of BOO (Miyata *et al*. 2019). BOO can increase systemic OS, with animal study results indicating that OS induced by bladder ischaemia or ischaemia/reperfusion affects bladder function (Lin *et al*. 2011). Moreover, activation of the NRF2/ARE pathway may attenuate bladder dysfunction caused by BOO (Hsieh *et al*. 2016). In mice, inner mitochondrial membrane peptidase 2-like (*Immp2l*) gene mutations cause increased production of superoxide ions, leading to bladder dysfunction (Lu *et al*. 2008; Soler *et al*. 2010). Hence, mutant mice with a high bladder weight/body weight ratio and increased detrusor muscle activity can serve as a model for OS (Andersson 2018). Accordingly, in the present study, a model of BOO was established, and gene expression levels in bladder tissue were measured. Next, primary BSMCs were isolated and cultured to emulate the growth of these cells in vivo, and H_2_O_2_ treatment was used to simulate an OS environment for the exploration of the effects of OS on the bladder. Using these two approaches, BOO was investigated from both in vivo and in vitro perspectives.

OS induces EMT; previously, human epidermal keratinocytes exposed to H_2_O_2_ exhibited protein expression consistent with EMT (Fukawa *et al*. 2012). Similar results were obtained in renal tubular epithelial cells (Rhyu *et al*. 2005), and chromium-induced EMT is dependent on intracellular ROS in pulmonary epithelial cells (Green *et al*. 1987). Specifically, type II EMT is associated with tissue regeneration and fibrosis, and its initiation and sustained occurrence are dependent on inflammation-induced injury until the induced damage or infection is eliminated (Kalluri and Weinberg 2009). This process can be a double-edged sword, as it is initially beneficial to wound healing but will cause ECM deposition and fibrosis under conditions of long-term inflammation (Tennakoon *et al*. 2015). Furthermore, bladder obstruction significantly induces histological and molecular changes, and EMT may play an important role in the pathogenesis of certain cases of BOO (Iguchi *et al*. 2014). In this study, we measured the expression of marker genes during EMT and found that the epithelial protein E-cadherin was downregulated, whereas the mesenchymal protein N-cadherin was upregulated, confirming that OS affects the EMT process of primary BSMCs. Our results provide evidence for this effect at the cellular level. Nevertheless, future research is needed to elucidate the underlying mechanisms. However, further research is required to elucidate additional factors capable of regulating genes expression in BOO.

## Conclusions

The present study found that TGFβ2 expression was significantly upregulated in the bladder tissue of a rat model of BOO and demonstrated the important regulatory role of TGFβ2 in EMT in BSMCs. More specifically, the inhibition of TGFβ2 expression eliminated the changes in EMT marker expression levels induced by H_2_O_2_ in bladder smooth muscle. Our research findings indicated that TGFβ2 is an important regulatory factor in BOO, and manipulating the expression of TGFβ2 may be a promising therapeutic approach in BPH. However, given the key roles of TGFβ2 and its downstream molecules in fibrosis progression, further investigation into the downstream pathways remains warranted.

### Supplementary Information

Below is the link to the electronic supplementary material.

Supplementary file1. BSMCs were labelled with antibodies against α-smooth muscle actin (α-SMA, green). The nuclei were labelled with DAPI (blue) (TIFF 13497 KB)

Supplementary file2. Knockdown efficiency of TGFβ2 by siRNA in BSMCs (TIFF 30065 KB)

Supplementary file3. H_2_O_2_-induced changes in TGFβ2 expression (TIFF 31133 KB)

Supplementary file4 (XLSX 10 KB)

Supplementary file5 (XLSX 11 KB)

Supplementary file6 (XLSX 10 KB)
