# Supplementary figures and images for: TGFβ2 mediates oxidative stress–induced epithelial-to-mesenchymal transition of bladder smooth muscle
Source: In Vitro Cell Dev Biol Anim. 2024 Feb 26;60(7):793–804. doi: 10.1007/s11626-024-00864-9 (PMC11297077; doi:10.1007/s11626-024-00864-9)

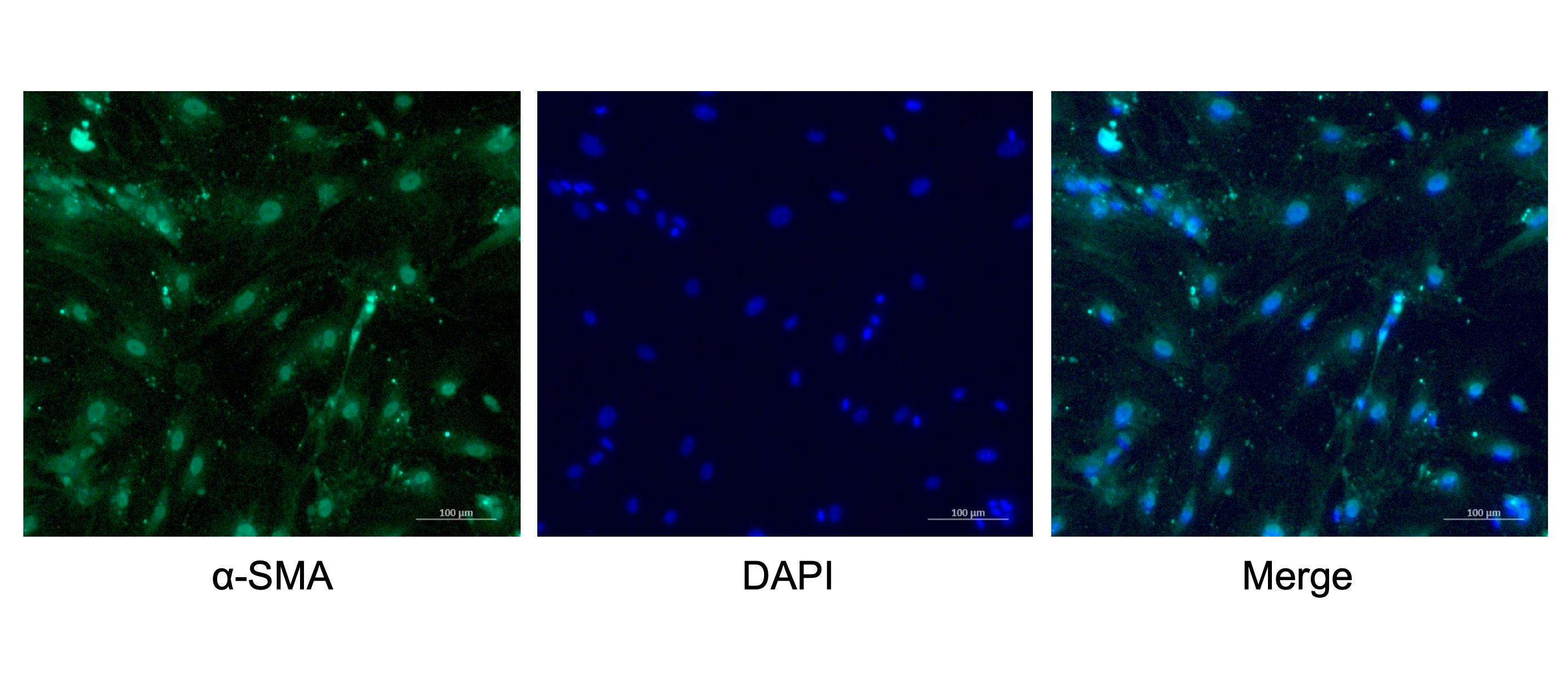

Supplement: Supplementary file 1 — Supplementary file1. BSMCs were labelled with antibodies against α-smooth muscle actin (α-SMA, green). The nuclei were labelled with DAPI (blue) (TIFF 13497 KB) [file 11626_2024_864_MOESM1_ESM.tiff]

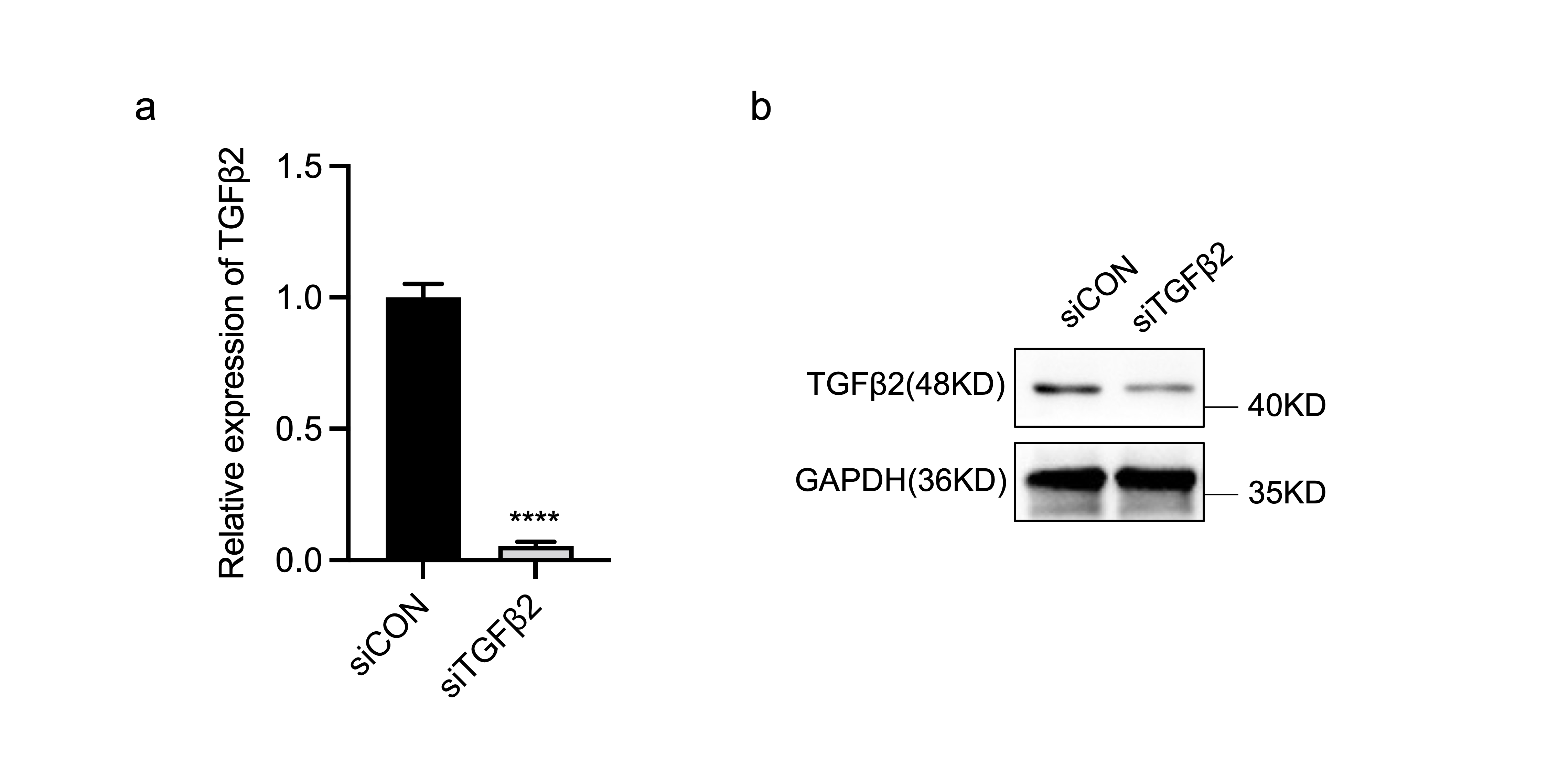

Supplement: Supplementary file 2 — Supplementary file2. Knockdown efficiency of TGFβ2 by siRNA in BSMCs (TIFF 30065 KB) [file 11626_2024_864_MOESM2_ESM.tiff]

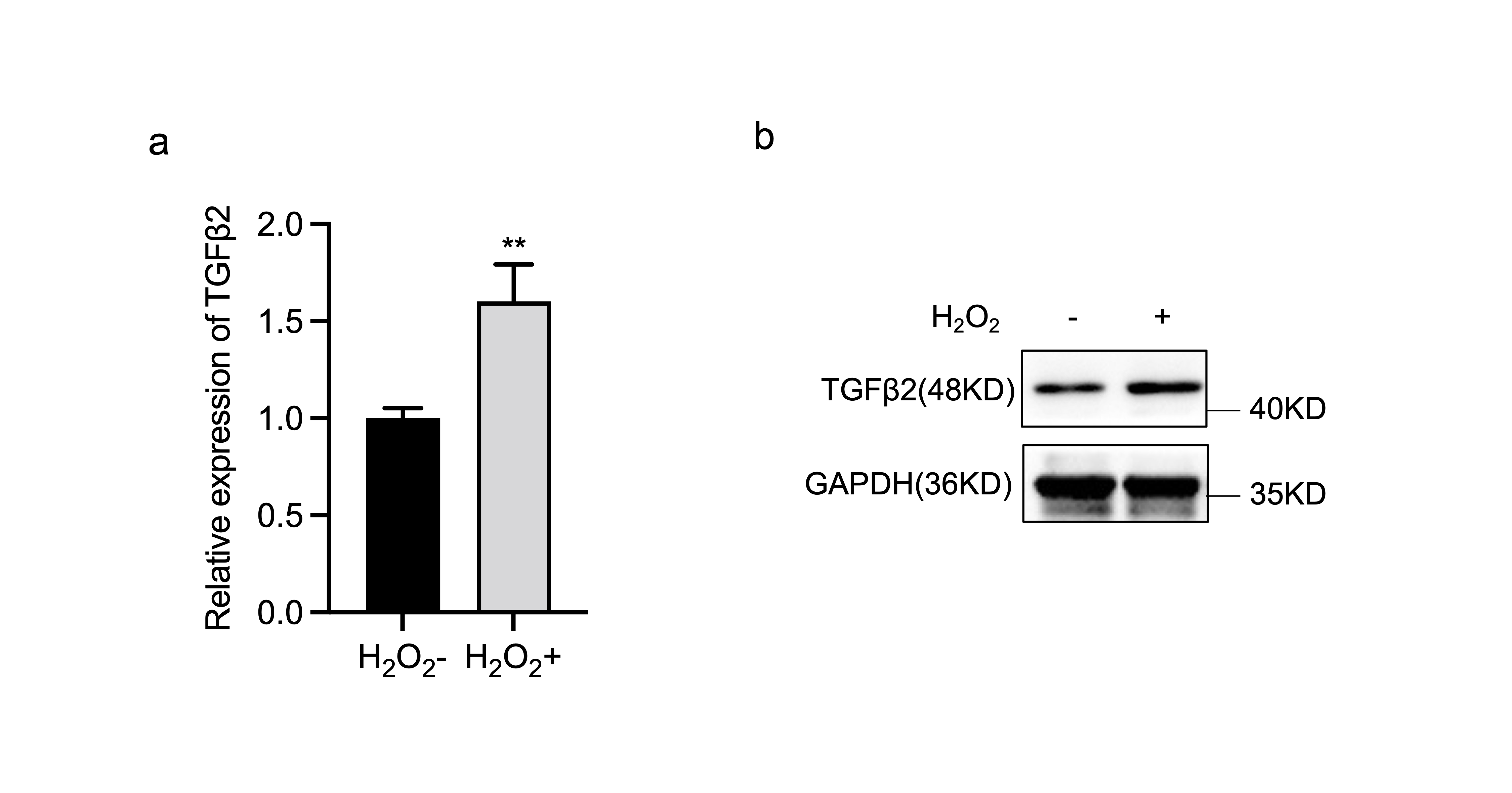

Supplement: Supplementary file 3 — Supplementary file3. H2O2-induced changes in TGFβ2 expression (TIFF 31133 KB) [file 11626_2024_864_MOESM3_ESM.tiff]
